# Anemia among Pediatric Critical Care Survivors: Prevalence and Resolution

**DOI:** 10.1155/2013/684361

**Published:** 2013-02-27

**Authors:** Quang N. Ngo, Doreen M. Matsui, Ram N. Singh, Shayna Zelcer, Alik Kornecki

**Affiliations:** ^1^Department of Pediatric Critical Care Medicine, Children's Hospital, London Health Sciences Centre, University of Western Ontario, 800 Commissioners Road East, P.O. Box 5010, London, ON, Canada N6A 5W9; ^2^Department of Pediatric Emergency Medicine, McMaster University, 1200 Main Street West, Hamilton, ON, Canada L8N 3Z5; ^3^Children's Health Research Institute, 800 Commissioners Road East, P.O. Box 5010, London, ON, Canada N6A 5W9; ^4^Centre for Critical Illness Research, 750 Base Line Road, Suite 300, London, ON, Canada N6C 2R5; ^5^Department of Hematology and Oncology, Children's Hospital, London Health Sciences Centre, University of Western Ontario, 800 Commissioners Road East, P.O. Box 5010, London, ON, Canada N6A 5W9

## Abstract

To determine the incidence of anemia among pediatric critical care survivors and to determine whether it resolves within 6 months of discharge. *Design*. A prospective observational study. Patients with anemia upon discharge from the pediatric critical care unit (PCCU) underwent in hospital and post hospital discharge followup (4–6 months) for hemoglobin (Hb) levels. *Setting*. A medical-surgical PCCU in a tertiary care center. *Patients*. Patients aged 28 days to 18 years who were treated in the PCCU for over 24 hours. *Measurements and Main Results*. 94 (24%) out of 392 eligible patients were anemic at time of discharge. Patients with anemia were older, median 8.0 yrs [(IQR 1.0–14.4) versus 3.2 yrs (IQR 0.65–9.9) (*P* < 0.001)], and had higher PeLOD [median 11 (IQR 10–12) versus 1.5 (1–4) (*P* < 0.001)], and PRISM [median 5 (IQR 2–11) versus 3 (IQR 0–6) (*P* < 0.001)] scores. The Hb level normalized in 32% of patients before discharge from hospital. Of the 28 patients who completed followup, all had normalization of their Hb in the absence of medical intervention. *Conclusions*. Anemia is not common among patients discharged from the PCCU and recovers spontaneously within 4–6 months.

## 1. Introduction

Anemia is a frequent occurrence in critically ill patients [1] and red blood cell (RBC) transfusions are commonly administered [1–3]. However, adult and pediatric literature suggests that RBC transfusions in the critically ill pose multiple risks [4, 5] and are associated with worsened clinical outcomes [4–7]. Conversely, restrictive transfusion practices with subnormal levels of hemoglobin (Hb) are well tolerated by the critically ill [1, 2, 4] and are associated with improved patient outcomes when compared to a liberal transfusion strategy [8]. While this issue is largely unaddressed in the pediatric population, the only randomized controlled trial in children to date (TRIPICU trial) supports the use of a restrictive transfusion strategy in critically ill children [9]. 

 We postulated that pediatricians would demonstrate a conservative approach to the use of RBC transfusions which would result in a high incidence and magnitude of anemia among patients discharged from the pediatric critical care unit (PCCU). This phenomenon has recently been described in a population of adult intensive care survivors in whom the prevalence of anemia increased to 80% [10]. There is no published data regarding the occurrence of anemia in children after critical care discharge. The objective of this study was to test the hypothesis that anemia is common among patients discharged from the PCCU and to determine the duration of this anemia. 

## 2. Materials and Methods

The study protocol was reviewed and approved by the University of Western Ontario Health Sciences Research Ethics Board. Written informed consent for blood and data collection was obtained from each patient and/or their parent or guardian. 

A prospective observational study was conducted in two stages between September 2009 and November 2010 in the PCCU of a tertiary care, university-affiliated hospital. 

The first stage of the study was to establish the prevalence of anemia among patients at time of discharge from the PCCU. Anemia was defined as a Hb level 2 standard deviations below the mean adjusted value for age and sex (Table 1) [11]. Severe anemia was defined as Hb ≤ 7 g·dL^−1^ for patients ≤ 1 year of age and Hb ≤ 8 g·dL^−1^ for children ≥ 1 year of age. All patients discharged from the PCCU during the study period were screened for anemia. *Inclusion Criteria*. Patients 1 month–18 years of age, admitted to the PCCU for greater than 24 hours with documented HgB level on capillary, venous, or arterial blood sample collected within 48 hours of discharge from the PCCU were eligible for inclusion. *Exclusion Criteria.* Patients with cyanotic heart disease, chronic lung disease, chronic renal failure, preexisting chronic anemia, active bleeding, and patients undergoing myeloablative therapies were excluded from the study. 

The demographic data (age and sex), diagnosis, admission pediatric risk of mortality (PRISM III) score [12] and pediatric logistic organ dysfunction(PELOD) score [13] of each study patient were recorded at PCCU admission. Laboratory data collected included Hb on discharge, mean corpuscular volume (MCV), and reticulocyte count.

The second stage of the study was to establish the rate of resolution of the anemia, determined through in hospital and post hospital followup. All patients with anemia upon discharge from the PCCU who agreed to participate were followed during the remainder of their hospital stay and Hb levels, blood transfusions, and/or iron supplementation were documented until they were discharged. 

Patients with anemia on a blood sample drawn within 72 hours of discharge from hospital were eligible for a post hospital followup. The patients were required to visit the hospital 4–6 months after discharge. At that visit, a blood sample was drawn for laboratory measurement of Hb level, MCV, reticulocyte count, and ferritin levels. The parents and/or patients were interviewed by a research assistant to exclude the use of medications or the onset of new illnesses that might alter the body's response to anemia. Patients were excluded from the post hospital followup portion of the study if they were from remote areas that made followup at our institution impractical or if they received iron supplementation.

### 2.1. Statistical Analysis

Data were analyzed using SigmaStat software, version 3.5 for Windows (Systat Software, Inc., Chicago, IL). Normally distributed continuous variables were compared between two groups using the independent samples *t*-test, and the Mann-Whitney test was used for skewed continuous outcomes. A one-way ANOVA was used to compare differences in continuous variables for more than two groups. Data are expressed as means (standard deviation, SD) or when the data are skewed, as medians (25th–75th percentile). A *P* value <0.05 was considered statistically significant. 

## 3. Results

### 3.1. Demographics and Prevalence

**Figure 1:**
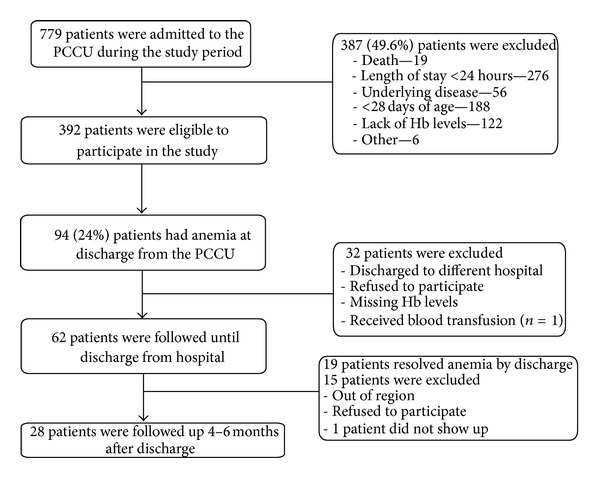
Flow chart of the study.

The flow chart of the study is presented in Figure 1. The patient demographics are presented in Table 2. During the study period (September 2009–November 2010), 779 patients were admitted to the PCCU (Figure 1). Three hundred and eighty seven patients (49.5%) were not eligible to participate in the study (e.g., chronic lung disease, known anemia, admission < 24 hours, age ≤ 28 days). Of the 392 patients eligible for the study, 94 (24%) patients had anemia upon discharge from the PCCU. Fifteen (3.8%) had severe anemia. 

### 3.2. Transfusion Details during the Study Period (in the PCCU)

**Figure 2:**
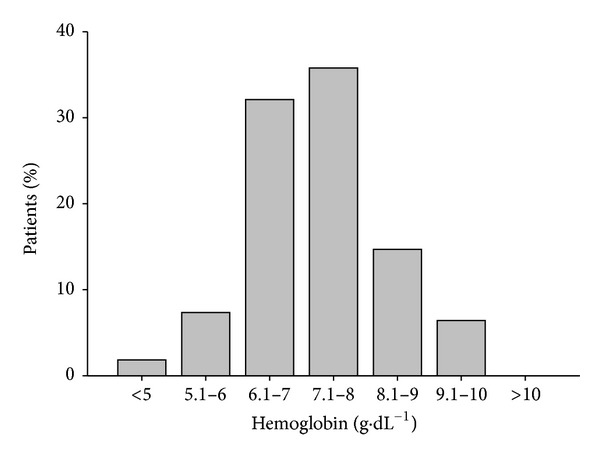
Percentage of patients receiving transfusions at predefined hemoglobin thresholds during study period.

During the study period, 74 of the study patients (18.8%), with a median age of 2.3 yrs (IQR 0.64–13.5), received 109 packed red blood cell transfusions. The mean pretransfusion Hb was 7.3 ± 1.1 g·dL^−1^. Forty-one percent (45/109) of the transfusions occurred below the Hb threshold of 7.0 g·dL^−1^ (Figure 2). 

### 3.3. Recovery from Anemia (Followup)

#### 3.3.1. In Hospital Followup

62 of the 94 (66%) patients with anemia upon discharge from the PCCU (Figure 1) participated in the followup until discharge from hospital. Thirty two patients were excluded because of the following: missing Hb levels (*n* = 19), discharge to a different hospital (*n* = 8), refusal to participate in the study (*n* = 3), or receipt of blood transfusions (*n* = 2) (Figure 1). The patient demographics are presented in Table 3. All had a normal MCV on admission and all experienced a drop in Hb during their PCCU stay. Sixteen out of the 62 (26%) patients received RBC transfusions during their stay in the PCCU. Nineteen (31%) recovered to normal Hb levels (without transfusion) while still in hospital after a median period of 16.5 days (IQR 11.0–27.5) after discharge from the PCCU, showing a recovery rate of 1.9 ± 0.74 g·dL^−1^ of Hb per week. These patients were discharged from hospital after a median of 28 days (IQR 18–51.5) compared to a median of 7 (IQR 4–11) days for patients in whom the anemia persisted at the time of discharge from hospital (*P* = 0.003).

#### 3.3.2. Post Hospital Followup

43 patients had anemia upon discharge from hospital. Twelve patients were out of region, resulting in 31 patients that were eligible for the long-term followup. Two (6%) refused participation in the study, resulting in 29 patients enrolled and 28 completing the study. All patients were found to have a normal Hb level within 4 to 6 months. Ferritin levels (54 ± 29.1 *μ*g·L^−1^) and MCV were also normal (84.5 ± 5.5 fL). None of the followup patients was transfused or received iron supplementation after discharge from hospital. 

## 4. Discussion

The results of this study did not support our main hypothesis. The incidence of anemia among patients discharged from the PCCU (24%) was lower than we expected and it had resolved in all followup participants within 6 months of PCCU discharge. 

The mean pretransfusion Hb level in our study group was 7.3 ± 1.1 g·dL^−1^ and over 80% of these patients were transfused at an Hb level ≤ 8 g·dL^−1^ (Figure 2). This is a lower threshold Hb level than observed in our PCCU (8.2 ± 1.8 g·dL^−1^ data not published) in the year 2000 and reported in other North American centers (8.8 ± 2.6 g·dL^−1^) [14] in 2000 and is consistent with the restrictive transfusion strategy currently promoted in the literature [9]. Regardless, in our current study only 24% of patients had anemia on discharge from the PCCU. This contrasts sharply with the reported rates of anemia in adult critical care survivors (50–77%) [10] and lower still than the 80% rate reported more recently in adults, suggesting that the incidence of anemia in adults has increased following the implementation of a restrictive transfusion strategy [15]. 

In our study patients with anemia were older and, as expected, more ill (higher PRISM and PELOD scores) compared to patients without anemia (Table 2). The association between severity of illness in the critical care population and anemia has been reported previously [1, 14, 16, 17]. We expected that the smaller blood volumes of younger children would make them particularly susceptible to anemia secondary to frequent blood sampling [16, 17] and that they would be disproportionately represented among patients discharged from the PCCU with anemia. Our study demonstrates the opposite and may reflect different thresholds for blood transfusion between infants and older children. 

During critical illness, the erythropoietic response to anemia is blunted by reduction in erythropoietin production [18, 19] and bone marrow suppression by various inflammatory cytokines [20–22]. Little is known regarding the rate of recovery of normal erythropoiesis following critical illness. Some suggest that the effect of blunted erythropoiesis may be prolonged because of the presence of inflammatory cytokines during recovery [23]. Other authors suggest that erythropoietin can be restricted by iron deficiency [24] and patients discharged from the PCCU may have depleted iron stores [18] and/or suffer from functional iron deficiency [25]. The rate of recovery of the anemia is important as anemia in general, and iron deficiency in particular, are known to impair immunity and increase infection risk [26], impair myocardial function [27], increase risk of thrombosis [28], cause fatigue [29], and alter behavioral and developmental function [30]. We found that the anemia resolved in less than 4–6 months in all patients that underwent post hospital followup (*n* = 28). Furthermore, 19 out of 62 patients (31%) with anemia on discharge from the PCCU recovered their Hb to normal levels while still in hospital. Since patients with anemia on discharge from the hospital were discharged significantly earlier than patients in whom the anemia resolved, one may speculate that most of the children may have shown resolution of their anemia well before the 4–6 month follow-up time. To the best of our knowledge, the rate of recovery of Hb in healthy children (e.g., after blood donation) has not been established. The rate of recovery in healthy adults is about 1.0 g·dL^−1^ per week [10]. This is significantly longer than the rate observed in our study (1.9 ± 0.74 g·dL^−1^ per week) among patients whose Hb recovered prior to hospital discharge (*n* = 20). Since Hb levels were not checked daily, this probably represents an underestimation of rate of recovery. A similar study in adults [23] showed a slower rate of recovery with anemia persisting in 53% of patients beyond 6 months. The slow recovery rate was attributed to chronic inflammation as the patients who did not recover had higher inflammatory markers (Il-6 and C-reactive protein). 

Although the determination of the etiology of the anemia was not within the scope of this study, the normal MCV and red cell distribution width on discharge exclude iron deficiency anemia [11] and the relatively fast resolution of anemia in children who stayed longer on the medical ward leads us to speculate that hemodilution may have played an important role. The vasodilatory effect of most sedative and analgesic drugs provided to children who required mechanical ventilation, the effect of positive pressure ventilation on preload requirement [31], and inappropriate antidiuretic hormone secretion may lead to hemodilution in the critically ill child. Unfortunately, the fluid balance for the patients who participated in the study was not available to support our hypothesis. 

This study is the first to examine the incidence and time to resolution of anemia among children discharged from the PCCU. However, there are several limitations to this study; specifically the relatively small number of patients who participated in the follow-up portion (*n* = 62) of the study, the relatively large number of patients who were excluded from the follow-up study, and the small number of infants. As serial measurements of Hb were not done, it was not possible to determine the time point at which anemia resolved in all patients. Generalizability of the study to other centers with a different patient mix or illness severity (specifically those with pediatric cardiac surgery patients) may be limited. 

## 5. Conclusions

We have shown that anemia was not very common among patients discharged from the PCCU in the era of a restrictive transfusion strategy. It was more common in older children and it self-resolved within 6 months. The rate of Hb improvement suggested a normal erythropoietic response. Further studies are needed in a larger group of children, particularly those in the younger age group and children with higher PRISM scores. The contribution of hemodilution to the magnitude of anemia in the critically ill pediatric patient should also be examined. 

## Figures and Tables

**Table 1:** Anemia as defined by hemoglobin (Hb) levels based on age (11).

Age	Hb (g·dL^−1^)
<2 m	<8.0
3–11 m	<9.0
1–4 y	<10.0
5–13 y	<11.0
Females ≥ 14 years	<12.0
Males ≥ 14 years	<13.0

**Table 2:** Patient demographics.

	Total patients	Nonanemic	Anemic	*P* value*
No.	779	298	94	—
Age (years)	1.45 (0.8–9.4)	3.2 (0.65–9.9)	8.0 (1.0–14.4)	<0.001
Female (%)	367 (47%)	148 (50%)	49 (52%)	—
PRISM	3 (0–7)	3 (0–6)	5 (2–11)	<0.001
PELOD admission	10 (0–11)	2 (0–11)	10 (0–11)	0.002
PELOD max	10 (1–11)	2 (1–11)	11 (10–12)	<0.001
Length of PCCU stay (days)	2 (1–4.3)	1 (1–4)	4 (1.4–11)	<0.001
Discharge Hb (g/dL)	12.0 ± 3.0	11.7 ± 1.6	8.4 ± 1.6	<0.001

Data are presented as median (25th–75th percentiles) or mean ± SD.

*Anemic versus nonanemic.

**Table 3:** Follow-up patient characteristics (*n* = 62).

Age (years)	13.7 (4.4–16.4)
Under 2 years	11 (18%)
Sex (female)	23 (47%)
Diagnosis *N* (%)	
Postoperative	26 (42)
Trauma	16 (26)
Respiratory failure	9 (14)
Sepsis	9 (15)
Others	2 (2)
Length of stay PCCU (days)	3.0 (2.0–9.0)
Length of stay hospital (days)	11.0 (5.5–26.0)
PRISM at admission	5.0 (2.0–7.5)
PELOD at admission	7.0 (1–11)
PELOD max	11.0 (1–14)
Hb at admission (g/dL)	11.5 ± 1.8
Mean corpuscular volume at admission (fL)	84.1 ± 4.9
Hb at discharge from the PCCU (g/dL)	8.1 ± 1.8
Hb at discharge from hospital (g/dL)	10.6 ± 1.7

Data are presented as median (25th–75th percentile) or mean ± SD.
